# Never in mitosis gene A-related kinase-8 promotes proliferation, migration, invasion, and stemness of breast cancer cells via β-catenin signalling activation

**DOI:** 10.1038/s41598-023-32631-3

**Published:** 2023-04-26

**Authors:** Eunji Kang, Hong-Kyu Kim, Han-Byoel Lee, Wonshik Han

**Affiliations:** 1 Cancer Research Institute, Seoul National University, 101 Daehak-ro, Jongno-gu, Seoul, Republic of Korea; 2 Department of Surgery, Seoul National University College of Medicine, 101 Daehak-ro, Jongno-gu, Seoul, Republic of Korea; 3 Biomedical Research Institute, Seoul National University Hospital, 101 Daehak-ro, Jongno-gu, Seoul, Republic of Korea

**Keywords:** Cancer, Cell biology, Genetics, Stem cells, Oncology

## Abstract

Never in mitosis gene A (NIMA)-related kinase-8 (NEK8) is involved in cell cycle progression, cytoskeleton development, and DNA damage repair. However, its role in breast cancer has not yet been explored. To investigate this, *NEK8* was knocked down in MDA-MB-231, BT549, and HCC38 breast cancer cell lines. We observed a decrease in cell proliferation and colony formation owing to regulation of the G1/S and G2/M transitions. Furthermore, the expression of several cell cycle regulatory proteins was altered, including that of cyclin D1, cyclin B1, CDK4, CDK2, and surviving. *NEK8* knockdown impaired cell migration and invasion as well as reduced the expression of epithelial-mesenchymal transition markers. Regarding stem-cell characteristics, *NEK8* knockdown decreased the tumour sphere formation, aldehyde dehydrogenase activity, and stem-cell marker expression, including that of CD44, Sox2, Oct4a, and Nanog. Further analysis revealed that NEK8 interacts with β-catenin. Also, *NEK8* knockdown promoted β-catenin degradation. NEK8-silenced MDA-MB-231 cells inhibited xenograft tumour growth, metastasis, and tumour initiation in vivo. Using the Oncomine and TNMplot public databases, we found a significant correlation between NEK8 overexpression and poor clinical outcomes in breast cancer patients. Thus, NEK8 may be a crucial regulator of breast cancer progression and a potential therapeutic target.

## Introduction

Globally, breast cancer is the primary cause of cancer-related deaths in women accounting for approximately 30% of all cancer cases in women^1^. Despite remarkable advances in therapeutic strategies, distant metastasis and relapse remain to be overcome^2,3^. In fact, metastasis is the primary cause of morbidity and mortality among patients with cancer, accounting for 90% of breast cancer-related deaths^4–6^. It involves a complicated cascade of events, including cancer cell migration/invasion, epithelial-mesenchymal transition (EMT), and cancer cell stemness^7–9^. Therefore, information on the molecular mechanisms regulating metastasis and cancer recurrence is required to develop effective therapies to improve patient outcomes. In breast cancer cells, the activation of the Wnt/β-catenin pathway plays a crucial role in regulating the EMT and promoting self-renewal^10,11^. Thus, anomalous activation of the Wnt/β-catenin signalling pathway facilitates cell proliferation and cancer stem-cell (CSC) renewal and plays a crucial role in tumourigenesis and therapy response^11–13^.

Approximately 50% of patients with breast cancer exhibit abnormal Wnt/β signal activation^14–17^. The Wnt/β-catenin pathway regulates various cellular processes, including cancer cell proliferation, differentiation, and migration; CSC renewal; and carcinogenesis, which are associated with the development, recurrence, metastasis, and treatment response of tumours^12,18–20^. Aberrant Wnt/β-catenin signal activation results in the translocation of β-catenin to the nucleus, where it upregulates the transcription of several genes^21,22^. Activity of β-catenin is regulated through its phosphorylation, particularly at Ser33/Ser37/Thr41, by glycogen synthase kinase 3β, which leads to β-catenin ubiquitination and degradation^23^.

In addition, β-catenin is phosphorylated at Ser552 by Akt and PKA, which leads to an increase in its transcriptional activity through its stabilisation and nuclear translocation^24^. Further, β-Catenin is capable of activating T-cell factor (TCF)/lymphoid enhancer factor (LEF) transcription factors, which induce the transcription of genes playing significant roles in regulating cancer cell proliferation, invasion, and metastasis^25,26^. Therefore, the activation of Wnt/β-catenin signalling plays an important role in breast cancer progression^23,27^.

Never in mitosis gene A (NIMA)-related kinase 8 (NEK8) is a member of a family of serine/threonine protein kinases that are involved in cell cycle regulation, DNA damage response, mitosis, and alternative splicing^28^. Subsequently, alternative splicing regulates the EMT and stemness of cancer cells^29^. Also, cell cycle-related proteins play an important role in the proliferation, invasion, and drug resistance of various types of tumours^30–32^.

According to our ongoing research and an analysis of public databases, there is a significant upregulation in *NEK8* expression in breast cancer cells^33^. Expectedly, *NEK8* expression in breast cancer tissues is higher than that in normal breast tissues^34^. The malfunction of NEK8 is linked to the progression and development of various types of cancer^35–37^. Additionally, knockdown of NEK2 expression in breast and cervical cancers is associated with decreased β-catenin signalling, which regulates tumourigenesis, especially during abnormal tumour proliferation ^38,39^. Hence, targeting NEK8 could be a promising approach for breast cancer treatment. Nevertheless, the precise mechanisms by which NEK8 modulates the activation of β-catenin still need to be fully elucidated.

To our knowledge, this is the first study on the function of NEK8 in breast cancer cell proliferation, migration, invasion, and stemness. Our findings suggest that NEK8 functions as an oncogene via the regulation of the Wnt/β-catenin signalling pathway.

## Results

### NEK8 expression is upregulated in invasive breast carcinoma and is associated with poor clinical outcomes

To determine the clinical relevance of NEK8 in breast cancer, we evaluated *NEK8* mRNA expression patterns using data from the Oncomine, Gene Expression Profiling Interactive Analysis (GEPIA), and TNMplot databases.

**Figure 1:**
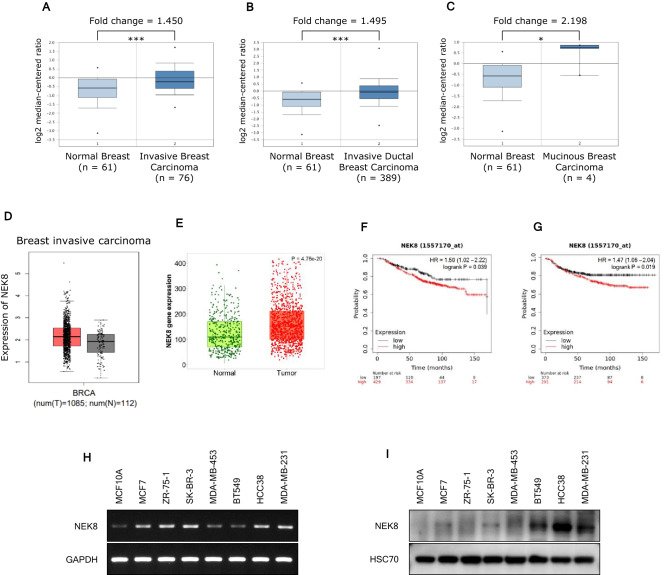
Increased never in mitosis gene A-related kinase-8 (*NEK8*) expression is associated with poor survival in patients with breast cancer. (**A**) *NEK8* mRNA expression in normal breast tissues and invasive breast carcinomas from the Oncomine database. (**B**) *NEK8* mRNA expression in normal breast tissues and invasive ductal breast carcinoma from the Oncomine database. (**C**) *NEK8* mRNA expression in normal breast tissues and mucinous breast carcinoma from the Oncomine database. Error bars, standard deviation; ***P < 0.001, **P < 0.01, *P < 0.05. (**D**) *NEK8* mRNA expression determined using RNA-sequencing in normal and tumour tissues from TNMplot.com. (**E**) *NEK8* expression in breast invasive carcinoma and normal tissues from the Gene Expression Profiling Interactive Analysis (GEPIA) database. Kaplan–Meier curves indicating (**F**) overall survival (OS) and (**G**) distant metastasis-free survival (DMFS) of patients with breast cancer were produced using the Kaplan–Meier plotter. (**H**) *NEK8* mRNA level in breast cancer and normal cell lines. GAPDH was used as a loading control. (**I**) NEK8 protein expression in breast cancer and normal cell lines. HSC70 was used as a loading control. The blots were cut prior to incubation with antibodies.

In the Oncomine breast cancer datasets, *NEK8* mRNA expression was 1.450-fold higher in invasive breast carcinoma samples than in normal tissues (*P* = 1.34e−5) (Fig. 1A). *NEK8* mRNA expression was 1.495-fold higher in invasive ductal breast carcinoma samples than in normal tissues (*P* = 8.43e−8) (Fig. 1B). In particular, *NEK8* mRNA expression was 2.198-fold higher in mucinous breast carcinoma samples than in normal tissues (*P* = 0.018) (Fig. 1C).

In the breast invasive carcinoma database from GEPIA, *NEK8* mRNA expression was significantly higher in breast cancer tissues than in normal tissues (Fig. 1D). The TNMplot data revealed that *NEK8* mRNA expression was higher in tumours than in normal tissues (*P* = 4.76e−20; Fig. 1E). Additionally, we examined the correlation between the expression of *NEK8* and patient survival with various types of cancer using Kaplan–Meier plotter^40^.

The screening criteria for the plotter were as follows: (1) breast cancer, (2) gene symbol: *NEK8*, (3) Affy ID: 1557170_at, (4) survival: overall survival (OS) and distant metastasis-free survival (DMFS), and (5) use of an earlier release of the database: 2017 (n = 5143). Kaplan–Meier survival curve analysis showed that increased *NEK8* mRNA expression was negatively associated with OS (hazard ratio [HR] = 1.50, *P* = 0.039; Fig. 1F) and DMFS (HR = 1.47, *P* = 0.019; Fig. 1G).

To identify differential *NEK8* mRNA expression, we examined a panel of breast cancer cell lines and the normal breast epithelial cell line MCF10A. Results from RT-PCR and western blotting analysis showed an upregulation of *NEK8* mRNA (Fig. 1H) and protein expression (Fig. 1I) in breast cancer cell lines compared to the normal breast epithelial cell line, MCF10A.

### NEK8 silencing suppresses proliferation and induces cell cycle arrest in breast cancer cells

**Figure 2:**
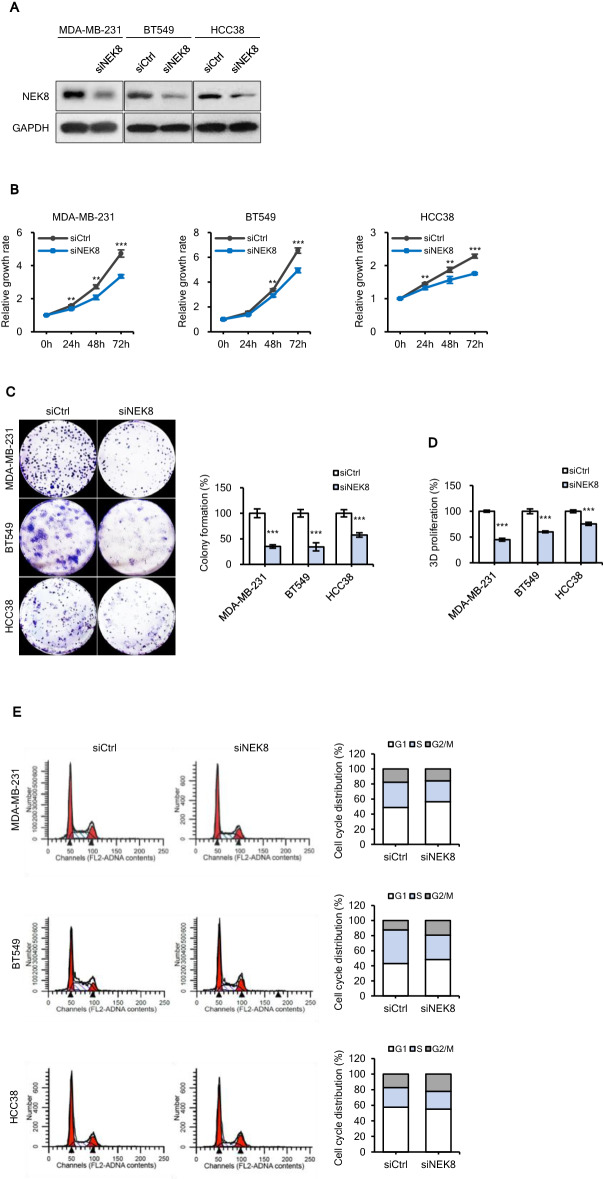

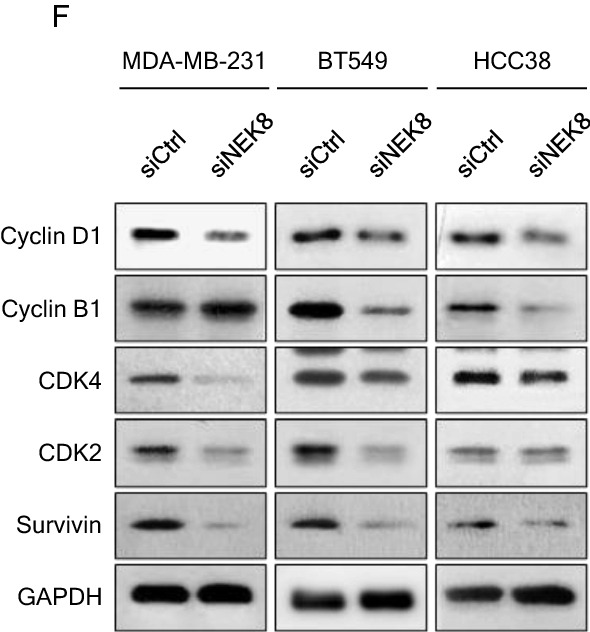
Knockdown of never in mitosis gene A-related kinase-8 (NEK8) attenuates proliferation and induces cell cycle arrest in breast cancer cells. MDA-MB-231, BT549, and HCC38 cells were transfected with control (siCtrl) or NEK8-silencing (siNEK8) small interfering RNA. (**A**) On day 3 after transfection, western blotting was used to confirm the knockdown of NEK8 in the siNEK8 group compared with that in the siCtrl group. (**B**) NEK8 knockdown inhibited the proliferation of breast cancer cells after 24, 48, and 72 h. The graph is representative of at least three independent experiments (n ≥ 3), where each experiment was performed in triplicate. Error bars, standard deviation (SD); ***P < 0.001, **P < 0.01, *P < 0.05. (**C**) NEK8 knockdown decreased colony formation. Representative images at least three independent experiments that showed similar results of colony growth (n ≥ 3). (**D**) In the 3D Matrigel matrix, cells with NEK8 knockdown showed reduced proliferation after 5 days. Error bars, SD; ***P < 0.001, **P < 0.01, *P < 0.05. (**E**) Distribution of cell cycle phases measured using flow cytometry. (**F**) Expression of cell cycle-related genes was confirmed using western blotting 72 h after NEK8 knockdown. GAPDH was used as a loading control. The figure is representative of at least three independent experiments that showed similar results (n ≥ 3). The blots were cut prior to incubation with antibodies.

To elucidate the function of NEK8 in the modulation of breast cancer cell proliferation, we used small interfering RNA (siRNA)-directed knockdown to deplete NEK8 expression in breast cancer cell lines overexpressing NEK8. After siRNA transfection into MDA-MB-231, BT549, and HCC38 cells, NEK8 protein expression was evaluated via western blotting (Fig. 2A).

The CellTiter-Glo assay results revealed that NEK8 silencing reduced the survival rate of MDA-MB-231, BT549, and HCC38 cells compared with that of the controls (Ctrl) (Fig. 2B). Furthermore, NEK8 silencing suppressed the colony-formation ability of MDA-MB-231, BT549, and HCC38 cells (Fig. 2C).

We evaluated the impact of NEK8 knockdown on breast cancer cell proliferation in a 3D culture system that mimics tumour growth in vivo. As shown in Fig. 2D, NEK8 depletion significantly inhibited growth in 3D cultures. Also, cell cycle arrest was prominent in breast cancer cells transfected with NEK8 siRNAs. Following transfection with NEK8 siRNA, we observed a noticeable increase in the proportion of MDA-MB-231 cells in the G1 phase, and BT549 and HCC38 cells in the G2/M phase, compared to the control-silenced (siCtrl) groups.

NEK8 knockdown induced cell cycle arrest in the G1 and G2/M phases in the tested cell lines (Fig. 2E). Thus, to examine the role of NEK8 in cell cycle regulation in breast cancer, we investigated the effect of NEK8 knockdown on the expression of cell cycle-related genes. As shown in Fig. 2F, NEK8 knockdown downregulated the expression of cyclin D1, CDK4, CDK2, and survivin in MDA-MB-231 and BT549 cells. Furthermore, NEK8 knockdown decreased cyclin B1, CDK2, and survivin expression in HCC38 cells. Using RT-PCR to measure *cyclin D1*, *cyclin B1*, *CDK4*, *CDK1*, and *CDC25C* mRNA levels, we found that these genes are sensitive responders to NEK8 manipulation (Supplementary Fig. S1). NEK8 knockdown decreased the expression of *Cyclin D1, CDK4, Vimentin, Snail, Slug, SOX2, Nanog* in MDA-MB-231 cells. NEK8 knockdown decreased the expression of *Cyclin D1, CDK4, CDK1, Vimentin, Snail, Slug, Nanog* in BT549 cells. NEK8 knockdown decreased the expression of *Cyclin D1, Cyclin B1, CDK4, CDK1, CDC25C, Vimentin, Snail, Slug, Nanog* in HCC38 cells.

### NEK8 knockdown impairs the migration and invasion of breast cancer cells

**Figure 3:**
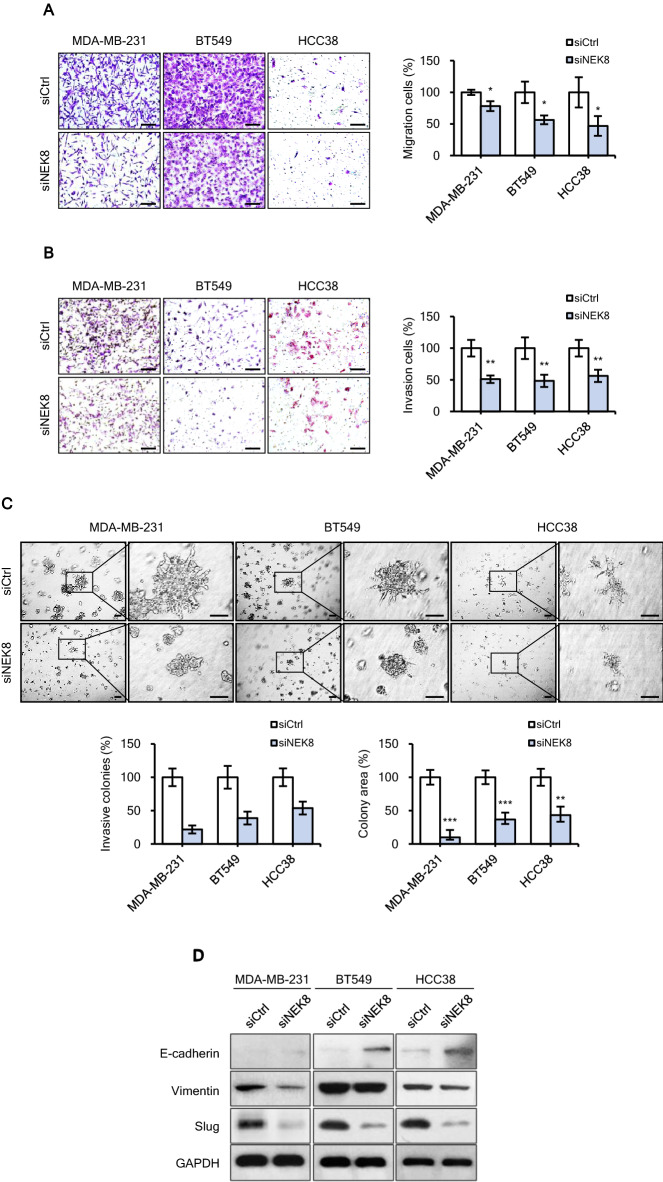
Knockdown of never in mitosis gene A-related kinase-8 (NEK8) inhibits the migration and invasion of breast cancer cells. (**A**) Migration and (**B**) invasion of MDA-MB-231, BT549, and HCC38 cells determined using Transwell assays (×200 magnification; scale bar, 50 μm). (**C**) Cells were grown on 3D Matrigel for 5 days; then, branching structures were counted using phase-contrast microscopy (×40 magnification; scale bar, 100 μm). The graph is representative of at least three independent experiments (n ≥ 3), where each experiment was performed in triplicate. Error bars, standard deviation; ***P < 0.001, **P < 0.01, *P < 0.05. (**D**) Expression of the epithelial-mesenchymal transition‑related proteins E‑cadherin, vimentin and Slug using western blotting. GAPDH was used as a loading control. The figure is representative of at least two independent experiments (n ≥ 2). The blots were cut prior to incubation with antibodies.

To investigate the role of NEK8 in metastasis, we compared the migration and invasion of the siCtrl group with those of the NEK8-silenced (siNEK8) group. The results showed that NEK8 silencing inhibited both the migration and invasion of MDA-MB-231, BT549, and HCC38 cells (Fig. 3A,B). Cells that are more invasive have been reported to form increased numbers of branches in Matrigel 3D cultures^41^. In Fig. 3C (upper picture), MDA-MB-231, BT549, and HCC38 cells in the Ctrl group exhibited invasive branching. The number and area of invasive colonies significantly decreased following NEK8 knockdown (Fig. 3C). Next, we assessed the impact of NEK8 on the expression of EMT markers in breast cancer cells. NEK8 knockdown significantly reduced the expression of vimentin, a mesenchymal marker, in MDA-MB-231, BT549, and HCC38 cells and induced the expression of E-cadherin, an epithelial marker, in BT549 and HCC38 cells (Fig. 3D).

### NEK8 knockdown impairs tumour sphere formation, aldehyde dehydrogenase 1 (ALDH1) activity, and cisplatin resistance

**Figure 4:**
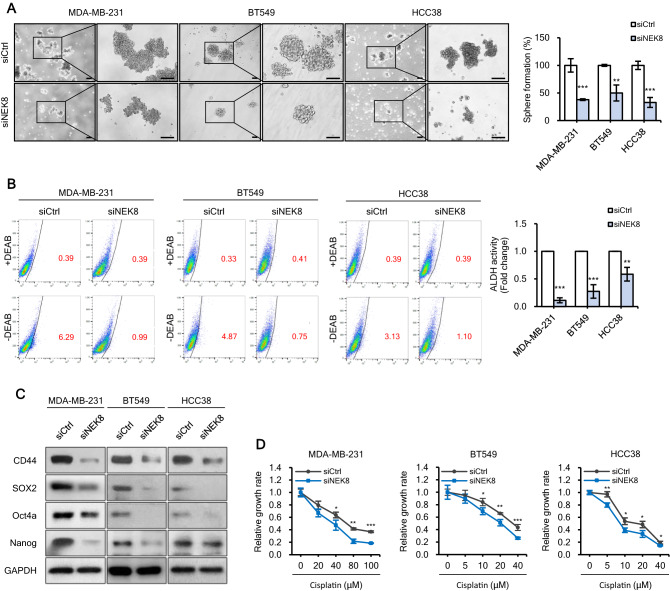
Knockdown of never in mitosis gene A-related kinase-8 (NEK8) inhibits tumour sphere formation, aldehyde dehydrogenase 1 (ALDH1) positive sub-populations, and drug resistance. (**A**) Representative images of spheres induced by culturing MDA-MB-231, BT549, and HCC38 cells treated with siCtrl (control) or NEK8-silencing (siNEK8) in mammosphere medium. NEK8 knockdown inhibited sphere formation in MDA-MB-231, BT549, and HCC38 cells (×40 magnification; scale bar, 100 μm). n = 3; Error bars, standard deviation (SD); ***P < 0.001, **P < 0.01, *P < 0.05. (**B**) Stemness in MDA-MB-231, BT549, and HCC38 cells was assessed with a ALDEFLUOR assay. Cell populations that disappeared in the presence of the specific ALDH inhibitor diethylaminobenzaldehyde (DEAB) were gated as ALDH^high^ cells. (**C**) Expression of cancer stem-cell-related proteins CD44, SOX2, Oct4a, and Nanog upon the knockdown of NEK8 in breast cancer cells. GAPDH was used as a loading control. The figure is representative of at least two independent experiments (n ≥ 2). The blots were cut prior to incubation with antibodies. (**D**) NEK8 knockdown decreased resistance to cisplatin treatment. The graph is representative of at least two independent experiments (n ≥ 2), where each experiment was performed in triplicate. Error bars indicate SD; ***P < 0.001, **P < 0.01, **P < 0.05.

We investigated the influence of NEK8 on CSC-like properties. Tumour sphere formation and self-renewal are characteristics of cultured CSCs^42,43^. To evaluate the function of NEK8 in CSCs derived from breast cancer cell lines, including MDA-MB-231, BT549, and HCC38 cells, we enriched CSCs by culturing the cells in mammosphere media under non-adherent conditions to form unattached tumour spheres. NEK8 knockdown significantly decreased sphere formation in MDA-MB-231, BT549, and HCC38 cells (Fig. 4a).

Aldehyde dehydrogenase (ALDH) activity is a reliable marker of stemness^44,45^. Flow cytometry results revealed that the proportion of ALDH-positive cells considerably decreased after NEK8 knockdown (Fig. 4B). NEK8 knockdown also reduced the levels of the CSC markers CD44, Sox2, Oct4a, and Nanog (Fig. 4C), and impaired resistance to cisplatin (Fig. 4D).

### NEK8 knockdown inhibits activation and nuclear translocation of β-catenin

**Figure 5:**
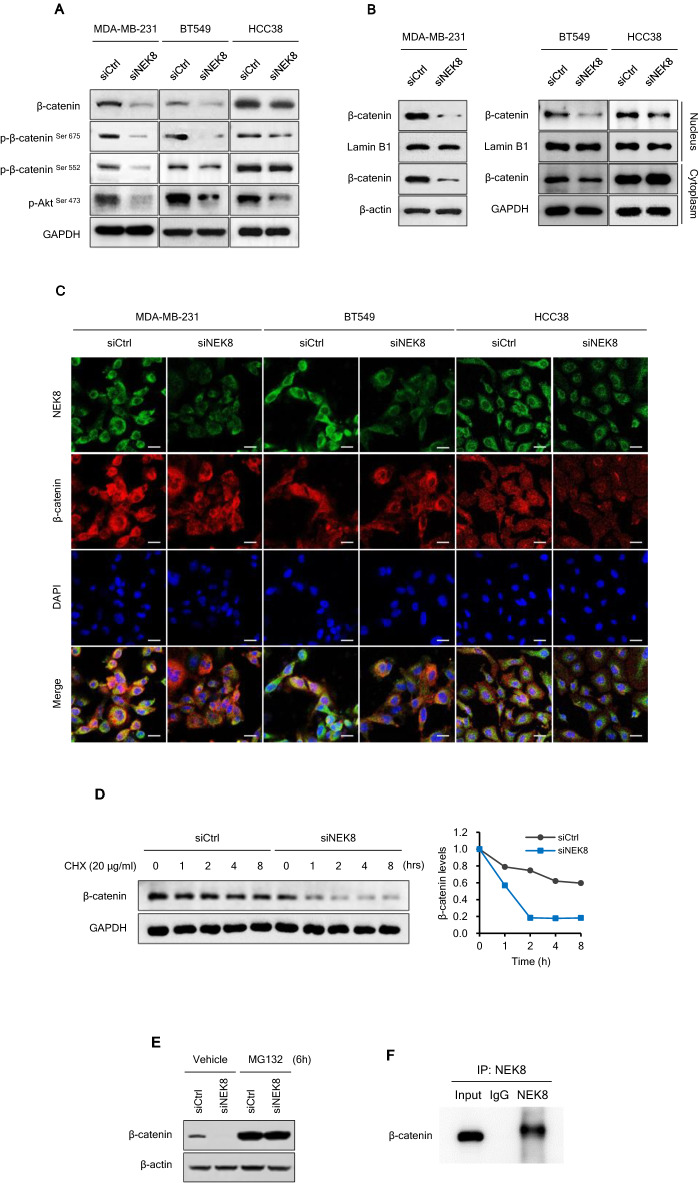
Knockdown of never in mitosis gene A-related kinase-8 (NEK8) inhibits β-catenin signalling in breast cancer cells. (**A**) The expression of total β-catenin, phospho-β-catenin^S675^, phospho-β-catenin^S552^, and phospho-AKT^S473^ in breast cancer cells transfected with NEK8-silencing (siNEK8) was detected using western blotting. (**B**) Representative western blot images demonstrating subcellular localisation of β-catenin in the nucleus and cytosol. (**C**) Confocal micrographs showing decreased nuclear β-catenin levels in MDA-MD-231, BT549, and HCC38 cells treated with siCtrl or siNEK8 for 72 h. GAPDH and β-actin were used as loading controls for whole cell lysates, and Lamin B was used as a loading control for nuclear fractions. The figure is representative of at least three independent experiments that showed similar results (n ≥ 3). (**D**) Stabilisation of β-catenin in NEK8 knockdown cells. The cells were treated with cycloheximide (CHX; 20 μg/mL) and collected at the indicated times for western blotting. (**E**) The cells were untreated or treated with MG132 (5 μM) for 6 h, and then subjected to western blotting. (**F**) Co-immunoprecipitation analysis of the interaction between NEK8 and β-catenin in MDA-MB-231 cells. The blots were cut prior to incubation with antibodies.

The β-catenin signalling pathway is crucial in controlling cancer-cell motility and EMT. After observing that NEK8 knockdown suppressed both EMT and cell motility, we then evaluated the effect of β-catenin signalling in MDA-MB-231, BT549, and HCC38 cells. The western blotting results revealed that NEK8 knockdown decreased phosphorylation of β-catenin at residues Ser675 and Ser552 (Fig. 5A). Phosphorylation of β-catenin via Akt promotes nuclear translocation and transcriptional activation of β-catenin, and thereby enhances cancer metastasis^46–48^.

NEK8 knockdown also decreased the phosphorylation of Akt at S473 (Fig. 5A). Subcellular fractionation revealed that siNEK8 reduced nuclear β-catenin levels. Consequently, β-catenin activation was significantly inhibited by siNEK8 (Fig. 5B). Using immunofluorescence, we observed that β-catenin accumulation in the cytoplasm and nucleus decreased in the siNEK8 group more than in the siCtrl group (Fig. 5C).

### NEK8 knockdown downregulates β-catenin expression by decreasing its stability

The RT-PCR results revealed that NEK8 knockdown reduced the mRNA levels of Wnt/β-catenin signalling target genes but had no effect on β-catenin mRNA levels (Supplementary Fig. S1). This indicates that post-transcriptional regulation is likely responsible for the decrease in β-catenin protein levels. To investigate the mechanism by which NEK8 controls β-catenin stability, we utilised cycloheximide to block protein synthesis and assessed the decay rate of β-catenin in the cells. The half-life of β-catenin decreased faster in siNEK8-treated MDA-MB-231 cells than in siCtrl cells (Fig. 5D).

Additionally, we treated the cells with the potent proteasome inhibitor MG132 to determine whether the stabilisation of β-catenin was due to the disruption of its ubiquitin-mediated proteasome degradation mechanism. As shown in Fig. 5E, decreased β-catenin expression was reversed by treatment with MG132 in NEK8-knockdown cells, showing that NEK8 knockdown resulted in the proteasomal degradation and the subsequent downregulation of β-catenin expression. Subsequently, the interaction between NEK8 and β-catenin was investigated using co-immunoprecipitation (Co-IP). Following NEK8 immunoprecipitation, β-catenin was detected in MDA-MB-231 cells using the corresponding antibodies (Fig. 5F). These results show that NEK8 interacts with β-catenin and that NEK8 knockdown promoted β-catenin degradation. Overall, our findings suggest that NEK8 has a direct impact on maintaining β-catenin stability and thereby, in activating Wnt/β-catenin signalling in breast cancer cells.

### NEK8 knockdown inhibits tumourigenesis and metastasis of MDA-MB-231 cells in vivo

**Figure 6:**
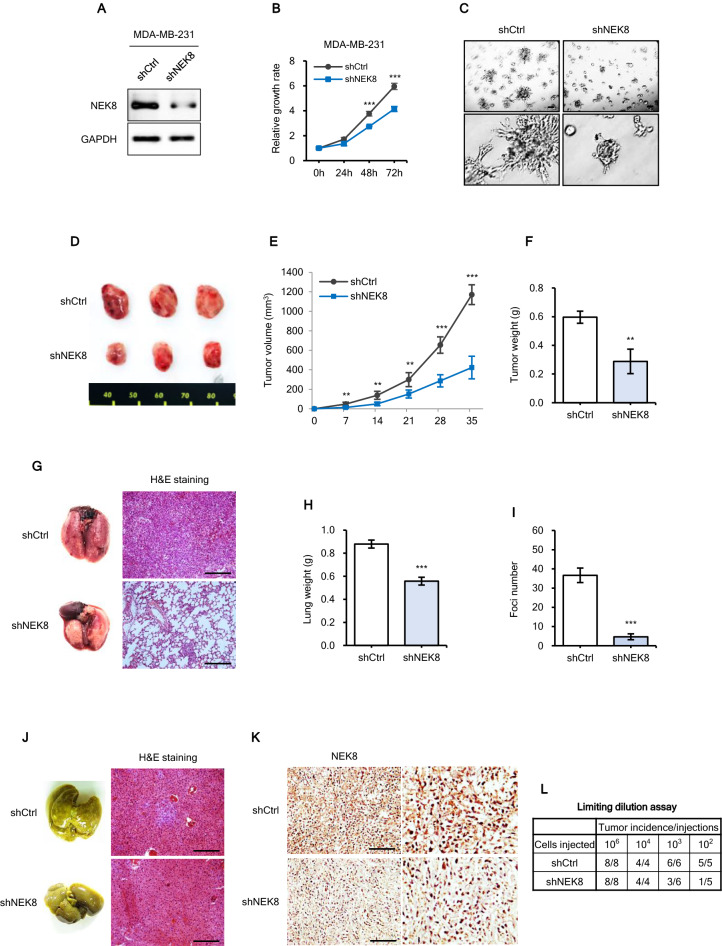

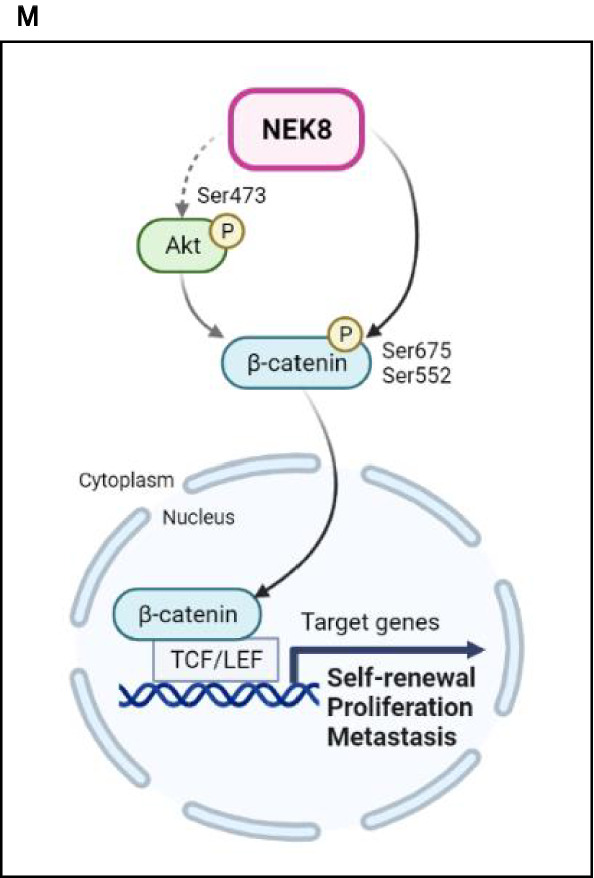
Never in mitosis gene A-related kinase-8 (NEK8) knockdown significantly inhibits tumour initiation and metastasis in MDA-MB-231 xenografts. (**A**) NEK8 expression in stable transfectants confirmed using western blotting. (**B**) Short hairpin RNA (shRNA) silencing of NEK8 attenuated MDA-MB-231 proliferation. (**C**) Phase-contrast images of 3D Matrigel growth of MDA-MB-231 cells with or without NEK8 knockdown (×40 magnification; scale bar, 100 μm). The results are representative of three independent experiments (n = 3). (**D**–**F**) Representative images and tumour sizes at 35 days after implantation showing that NEK8 knockdown decreased tumour volume and weight (n = 3). Images are representative of at least two experiments (n ≥ 2). (**G**) Representative haematoxylin and eosin (H&E) images of lung metastases. Scale bar = 50 μm. (**H**) Lung weights in the lung metastasis model. Values are presented as mean ± standard deviation (SD) (n = 3). ***P < 0.001. (**I**) Numbers of metastatic foci in the lungs. Values are presented as mean ± SD (n = 3). ***P < 0.001. (**J**) Representative H&E images of liver metastases. Scale bar, 50 μm. (**K**) Immunohistochemistry analysis to confirm the expression of NEK8 in tumours from either shCtrl or shNEK8 group. Scale bar, 10 μm. (**L**) Numbers of tumours generated and the results of the limiting dilution assay (10^6^, 10^4^, 10^3^, and 10^2^ cells). Numbers of tumours generated are shown in the table. (**M**) Schematic representation of the mechanism underlying the effect of NEK8 silencing on breast cancer cell proliferation, migration, invasion, and stemness mediated through the β-catenin signalling pathway. Created using BioRender (BioRender.com). NEK8 causes increased translocation of β-catenin to the nucleus through the phosphorylation of β-catenin. NEK8 also regulates the phosphorylation of AKT, which may affect the phosphorylation of β-catenin, as well as its activation and nuclear translocation, contributing to the enhancement of β-catenin transcriptional activity, which then results in increased self-renewal, proliferation, and metastasis of breast cancer cells.

After observing that NEK8 knockdown decreased the proliferation, migration, invasion, and stemness of cultured breast cancer cells, we assessed the functional effects of NEK8 in controlling tumour growth in a murine xenograft model. We knocked down NEK8 in MDA-MB-231 cells using short hairpin RNA (shRNA) (Fig. 6A).

NEK8 silencing inhibited cell proliferation in Matrigel 3D culture and attenuated the aggressive phenotype (Fig. 6B,C). We transplanted both control and NEK8-knockdown MDA-MB-231 cells into the mammary fat pads of female NOD.Cg-*Prkdc*^*scid*^* Il2rg*^*tm1Wjl*^/SzJ (NSG) mice (n = 3) and observed the growth of tumours for 35 days. Also, NEK8 knockdown significantly reduced tumour volume and weight (Fig. 6D–F).

The imaging results indicated that the rate of lung metastasis was lower in mice injected with shNEK8 cells than in those injected with shControl (shCtrl) cells. Additionally, lung weight was lower in the shNEK8 group than in the shCtrl group (Fig. 6H).

Haematoxylin and eosin (H&E) staining results revealed that the nodules on the surface of mouse lungs were metastatic. Histology confirmed that the number of both metastatic nodules and metastatic foci in the lungs (Fig. 6G,I) and liver (Fig. 6J) significantly decreased in the NEK8-knockdown group compared with those in the shCtrl group. The tumours were stained with NEK8 antibodies and the expression of NEK8 was found to be lower in the shNEK8 group than in the shCtrl group (Fig. 6K).

Furthermore, to determine the role of NEK8 in the promotion of stemness of breast cancer cells in vivo, we conducted xenograft experiments with MDA-MB-231 cells using a limited dilution xenograft assay. NOD/SCID mice inoculated with 10^4^, 10^3^, and 10^2^ NEK8-knockdown cells exhibited a considerably lower incidence of tumour initiation than mice inoculated with shCtrl cells (Fig. 6L).

## Discussion

Elevated expression of *NEK8* has been observed in primary breast tumours and NEK8 is associated with genetic instability and mutations^34^. NEK8 is highly expressed in various types of invasive breast cancer, and our findings indicate a link between high expression levels and poor OS in breast cancer patients, as suggested by analysis of public databases such as Kaplan–Meier plotter and the Oncomine database. Similarly, our ongoing research shows that *NEK8* expression is upregulated in primary breast tumours compared with that in normal breast tissues (manuscript under preparation).

NEK8 is a serine/threonine kinase that plays a crucial role in cell cycle progression^49,50^. Moreover, dysregulation or mutation of protein kinases has been reported to affect the genesis and progression of breast cancer^51^. Therefore, NEK8 overexpression is likely to induce the phosphorylation of proteins involved in cancer progression, namely those associated with proliferation, invasion, metastasis, and drug resistance. NEK2 is known to influence cancer progression by affecting the activation of pro-tumourigenic signalling pathways, such as Akt, Wnt/β-catenin or MAPK, in multiple types of cancers^52^.

Furthermore, it has been reported that NEK8 may potentially play the role of an oncoprotein in human gastric cancer through the HIF-1 signalling pathway^53^. A recent study using bioinformatic analyses has reported that NEK8 can function as a therapeutic target for gliomas^54^. However, only a few studies have investigated the function of NEK8 in breast cancer cells and its underlying molecular mechanisms. In this study, we found that NEK8 expression was higher in breast cancer cell lines than in normal cell lines.

NEK8 knockdown in breast cancer cell lines significantly inhibited cell proliferation, migration, invasiveness, stemness, and cisplatin chemoresistance in vitro. NEK8 regulated the expression of proliferation-related proteins, including cyclins and CDKs; EMT-related proteins, such as E-cadherin, vimentin, and Slug; and CSC markers, including CD44, Sox2, and Oct4. This indicated that NEK8 affects breast cancer metastasis by regulating the expression of genes related to proliferation, EMT, and stemness. Furthermore, the NEK8 knockdown via shRNA in MDA-MB-231 cells inhibited the tumour-initiating and metastatic potential of MDA-MB-231 cells in vivo.

Our findings support the hypothesis that increased NEK8 expression plays a crucial role in breast cancer cell growth and metastasis. WNT/β-catenin pathway plays a vital role in many cellular processes, including cell growth, differentiation, tumourigenesis, chemoresistance, and cell cycle progression. The translocation of β-catenin into the nucleus triggers various transcription factors, regulating the downstream signalling cascade. The WNT/β-catenin pathway is crucial for the maintenance of breast cancer cells, EMT, and stemness, making it a significant factor for the progression and invasion of breast cancer. Our research found that β-catenin is a crucial downstream mediator of NEK8 in breast cancer.

The downregulation of NEK8 led to a decrease in β-catenin phosphorylation at Ser552 and Ser675. Our Co-IP results indicated that NEK8 interacts directly with β-catenin, which implies that NEK8 is responsible for phosphorylating β-catenin at Ser552 and Ser675. This phosphorylation results in the stabilisation and nuclear accumulation of β-catenin, where it interacts with TCF/LEF-1 transcription factors and activates downstream target genes.

NEK8 knockdown also induced a decrease in the level of Akt phosphorylation at Ser473, which induced β-catenin phosphorylation at Ser552^47,55^ (Fig. 6M). Additionally, a previous study has reported the role of NEK8 in regulating breast cancer cell growth and mobility through the transcriptional coactivator with PDZ-binding motif (TAZ) pathway^56^. Another study has also demonstrated that TAZ and β-catenin expression are positively correlated and play a synergistic role in the invasion and metastasis of CSCs^21,57^. Furthermore, the Hippo‐YAP/TAZ signalling pathway has been confirmed to be located downstream of the standard Wnt signalling pathway, suggesting that NEK8 might be associated with the β-catenin-YAP/TAZ pathway.

Finally, this study has some limitations. Although our study demonstrates changes in β-catenin expression levels and subcellular locations, it does not prove a direct association between NEK8 and β-catenin. Therefore, future studies are required to confirm whether NEK8 interacts directly with β-catenin to induce phosphorylation and activation. Furthermore, complete details of the interaction between NEK8 and Akt were not obtained in this study. Thus, the mechanism underlying NEK8-induced Akt-mediated regulation of β-catenin phosphorylation requires further investigation.

Our study reveals new perspectives on the interplay between NEK8 and β-catenin in breast cancer cells, highlighting the crucial role of NEK8 in promoting malignancy through the activation of β-catenin signalling in breast cancer initiation and advancement.

## Methods

### Gene expression, survival, and prognosis analyses

*NEK8* mRNA expression levels were analysed using the Oncomine (https://www.oncomine.org) and TNMplot (http://www.tnmplot.com) databases. TNMplot with RNA-sequencing was used to evaluate *NEK8* mRNA expression in normal tissues. The association of *NEK8* expression with OS was determined using the Kaplan–Meier plotter database (http://kmplot.com/analysis/). Screening criteria for the plotter were as follows: (1) breast cancer, (2) gene symbol: *NEK8*, (3) Affy ID: 1557170_at, (4) survival: overall survival (OS) and distant metastasis-free survival (DMFS), and (5) use of an earlier release of the database: 2017 (n = 5143).

### Cell culture

The human breast cancer cell lines MCF-7, ZR-75-1, HCC38, and MDA-MB-231 were purchased from the Korean Cell Line Bank (Seoul, South Korea). The non-cancerous human breast epithelial cell line MCF10A and breast cancer cell lines SK-BR-3, MDA-MB-453, and BT549 were obtained from the American Type Culture Collection (Manassas, VA, USA). The cells were cultured in RPMI-1640 or Dulbecco’s modified Eagle medium (DMEM) (Biowest, Riverside, MO, USA) supplemented with 10% foetal bovine serum and 1% antibiotic–antimycotic (Gibco, Grand Island, NY, USA) under 5% CO_2_ in a humid atmosphere at 37 °C. The cell cultures were tested for contamination using a mycoplasma detection kit (MycoAlert, Lonza, Basel, Switzerland).

### NEK8 knockdown

MDA-MB-231 cells were transfected with non-targeting control siRNAs or NEK8-targeting siRNAs (Dharmacon Inc., Lafayette, CO, USA) using Opti-MEM and Lipofectamine RNA iMAX reagent (Invitrogen, Carlsbad, CA, USA) according to the respective manufacturer’s instructions.

To establish MDA-MB-231 cells with a stable NEK8 knockdown, cells were infected with NEK8 shRNA lentiviral particles (sc-61176-V, Santa Cruz Biotechnology, Santa Cruz, CA, USA) or non-targeting shRNA lentiviral particles (sc-108080, Santa Cruz Biotechnology). Information on the primers used is presented in Supplementary Table S3. The established cells were selected using puromycin (5 μg/mL) for over 3 weeks.

### Proliferation assay

Cell proliferation was measured using the Cell Titer Glo Luminescent Cell Viability Assay kit (Promega, Madison, WI, USA). Briefly, breast cancer cells (2 × 10^3^ cells/well) were seeded in 96-well plates after transfection with either NEK8-targeting siRNA or control siRNA.

After 24, 48, or 72 h, the cells were lysed with CellTiter-Glo reagent, and luminescence was measured using a Luminoskan Ascent luminometer (Thermo Fisher Scientific, Waltham, MA, USA).

### Migration and invasion assays

Breast cancer cells (5 × 10^4^) treated with siRNAs (siCtrl or siNEK8) were seeded in a Transwell plate with serum-free culture medium (Corning, Corning, NY, USA). The migrated cells were fixed with 4% paraformaldehyde, stained using crystal violet, and photographed under an inverted microscope (Nikon Eclipse Ci-L, Tokyo, Japan).

Invasion assays were performed in the same manner using a Matrigel-coated Transwell (Corning, Corning, NY, USA). In five random fields, the cells that were stained were counted, and the average number was determined.

### RT-PCR

Total RNA was extracted from breast cancer cells using the Qiagen RNeasy Mini kit (Qiagen, Hilden, Germany) in accordance with the manufacturer's instructions. After measuring the quality of the RNA using Nanodrop, total RNA (1 μg) was reverse transcribed to cDNA using a cDNA synthesis kit (Promega). cDNA was amplified using Taq DNA polymerase (Intron Biotechnology, Seongnam, South Korea) according to the manufacturer’s protocol. Information on the primers used is presented in Supplementary Table S3.

### Western blotting

For the isolation of total proteins, the cells were collected and lysed with radioimmunoprecipitation assay lysis buffer (Thermo Fisher Scientific). Nuclear and cytoplasmic proteins in the cells were extracted using a Nuclear Extraction kit (Abcam, Cambridge, UK) according to the manufacturer’s instructions.

Using the Pierce bicinchoninic acid Protein Assay kit (Thermo Fisher Scientific), the protein concentration was quantified, and the same amount of protein was loaded in 8%–12% sodium dodecyl sulfate–polyacrylamide gels. The separated proteins were transferred from the gels onto polyvinylidene difluoride membranes (Merck Millipore, Bedford, MA, USA). The membranes were incubated with the primary antibody overnight at 4 °C, and then with the secondary antibody for 1 h at room temperature (20–25 °C). The blots were cut prior to hybridisation with antibodies. The following primary antibodies were used: NEK8 (aviva OAAB10225, 1:1000), total β-catenin (CST 8480, 1:1000), cyclin D1 (CST 2978, 1:1000), Phospho-Akt (Ser 473) (CST 9271, 1:1000), vimentin (Calbiochem IF01, 1:1000), and GAPDH (Invitrogen MA5-15738, 1:1000). The secondary antibodies used were as follows: anti-rabbit (Bethyl A120-101P, 1:5000), anti-mouse (Bethyl A90-116P, 1:5000), anti-rabbit-fluorescein isothiocyanate (FITC) (Invitrogen, F-2765), and anti-mouse-phycoerythrin (Invitrogen, P-852). The protein bands were detected using Super Signal West Pico Chemiluminescent Substrate (Thermo Fisher Scientific).

### 3D Matrigel culture

For 3D culture, cells (2 × 10^3^ cells/well) were seeded in a plate coated with growth factor-reduced Matrigel matrix (BD Bioscience, San Diego, CA). The medium was replaced twice weekly. After 5 days, 3D cultured cell images were observed using an inverted microscope (Leica, Germany). The growth of cells was measured using the CellTiter-Glo^®^ 3D assay (Promega) following the manufacturer’s instructions.

### Mammosphere assay

MDA-MB-231, BT-549, or HCC38 cells (1 × 10^4^ cells/well) were plated in an ultra-low attachment well (Corning) with mammosphere medium: DMEM/F12 containing B27 (Gibco), insulin (Sigma, St. Louis, MO), EGF (20 ng/mL) (Peprotech, Rocky Hill, NJ, USA), bFGF (20 ng/mL) (Peprotech), LIF (Millipore, Temecula, CA, USA), hydrocortisone, and 1% antibiotic–antimycotic (Gibco). Mammospheres with diameters over 100 μm were counted after 7 days of culture.

### ALDEFLUOR assay

After NEK8 knockdown, ALDH activity in breast cancer cells was measured using an ALDEFLUOR assay kit (Stem Cell Technologies, Vancouver, Canada). The cells resuspended in ALDEFLUOR assay buffer containing ALDH substrate and diethylaminobenzaldehyde (DEAB) were incubated at 37 °C for 40 min. DEAB, a specific inhibitor of ALDH, served as the negative control. Flow cytometry data were analysed using FlowJo software (Treestar, San Carlos, CA, USA).

### Immunofluorescence

Cells grown on 8-chamber slides (ibidi, Munich, Germany) were washed in phosphate-buffered saline, fixed with 4% paraformaldehyde, permeabilised with Triton X-100, blocked in 5% bovine serum albumin for 1 h, and incubated with anti-NEK8 and β-catenin antibodies at 4 °C overnight. The cells were stained with secondary antibody conjugated with FITC or phycoerythrin for 1 h at room temperature (20–25 °C) and counterstained with 4′,6-diamidino-2-phenylindole (DAPI; BD Bioscience). The cells were imaged using a Zeiss 800 laser-scanning 800 confocal microscope (Carl Zeiss, Jena, Germany).

### Immunoprecipitation

To assess the interaction between NEK8 and β-catenin, immunoprecipitation was performed using an immunoprecipitation kit (Abcam) according to the manufacturer’s instructions. Cells were lysed in lysis buffer (300 μL) of supplemented with protease inhibitor, and then treated with NEK8 antibody overnight at 4 °C on a rotator. After incubation, the lysates were incubated with protein A/G sepharose for 1 h. A/G sepharose was washed with wash buffer, and the protein was eluted.

### Animal studies

All animal experiments were approved by the Seoul National University Institutional Animal Care and Use Committee under protocol #SNU 15112–3. Experiments were conducted in accordance with relevant guidelines, including the ARRIVE guidelines (https://arriveguidelines.org).

Briefly, 6–8-week-old female NSG mice (NOD/SCID/IL-2Rγnull; Jackson Laboratory, Bar Harbor, ME, USA) were xenografted with 10^6^ shRNA-transfected cells via subcutaneous inoculation into the mammary fat pad. Tumour growth was measured weekly using callipers, and volumes were calculated. Mice were euthanised after 35 days via CO_2_ inhalation. Tumours, lungs, and livers were harvested and fixed in 10% formalin (Biosesang, Seongnam, Korea).

Then, lungs and livers were prepared for paraffin sectioning and the sections were stained with H&E. The tumours were prepared for immunohistochemistry using the anti-NEK8 antibodies. For limiting dilution assays, serial dilutions (10^6^, 10^4^, and 10^2^) of either Ctrl or NEK8-knockdown MDA-MB-231 cells were injected into the mammary fat pads of NSG mice.

### Statistical analysis

Data are presented as mean ± standard deviation. Statistical analyses were performed using one-way analysis of variance. The differences between groups were analysed using Student’s *t*-test and *P* ˂ 0.05 was considered statistically significant.

## Supplementary Information

Supplementary Information 1.

Supplementary Tables.

Supplementary Legends.

Supplementary Figure S1.

## Data Availability

The data of this study are available within the paper and its supplementary information files, which are all available from the authors upon reasonable request. The datasets generated during and/or analysed during the current study are available from the corresponding author on reasonable request.
