# Supplementary material for: Never in mitosis gene A-related kinase-8 promotes proliferation, migration, invasion, and stemness of breast cancer cells via β-catenin signalling activation
Source: Sci Rep. 2023 Apr 26;13:6829. doi: 10.1038/s41598-023-32631-3 (PMC10133229; doi:10.1038/s41598-023-32631-3)
Supplement: Supplementary file 1 — Supplementary Information 1. [file 41598_2023_32631_MOESM1_ESM.docx]

**Original gels/blots**

**
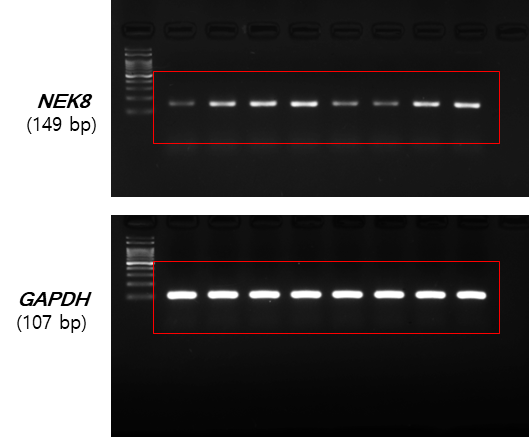
Figure 1H**

**Figure 1I**

**
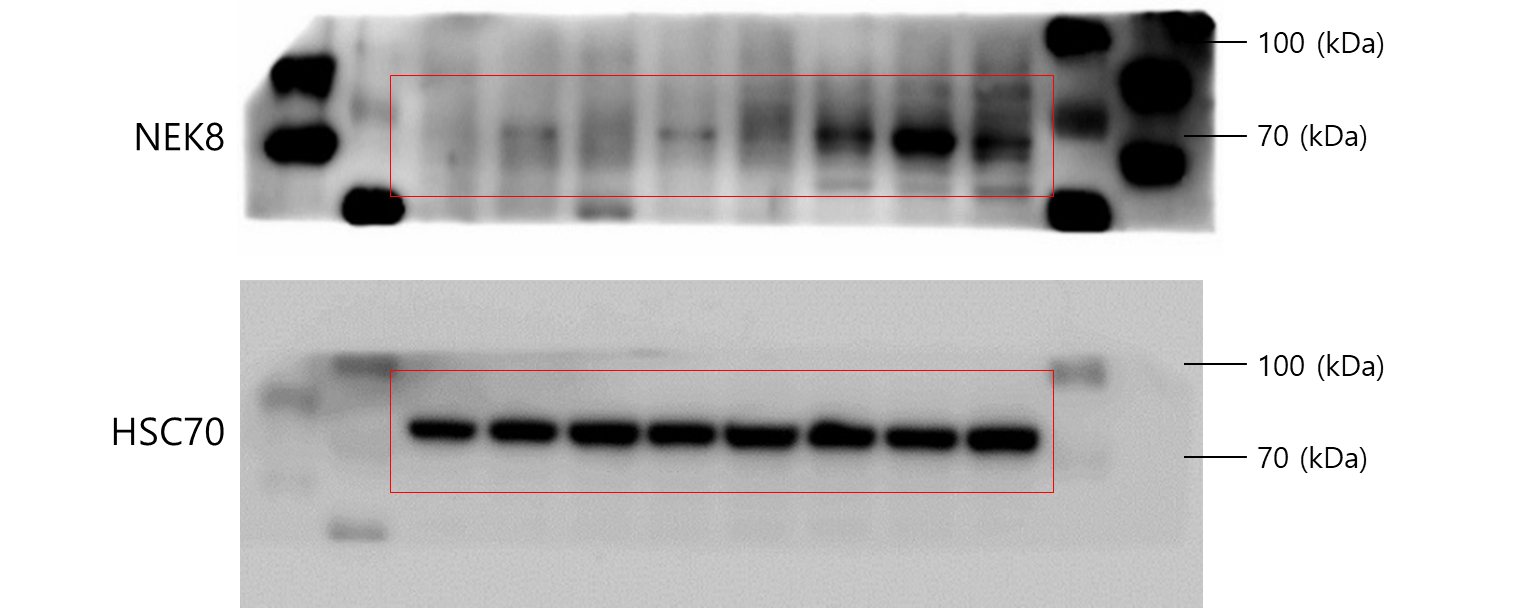
**

**Figure 2A**

**
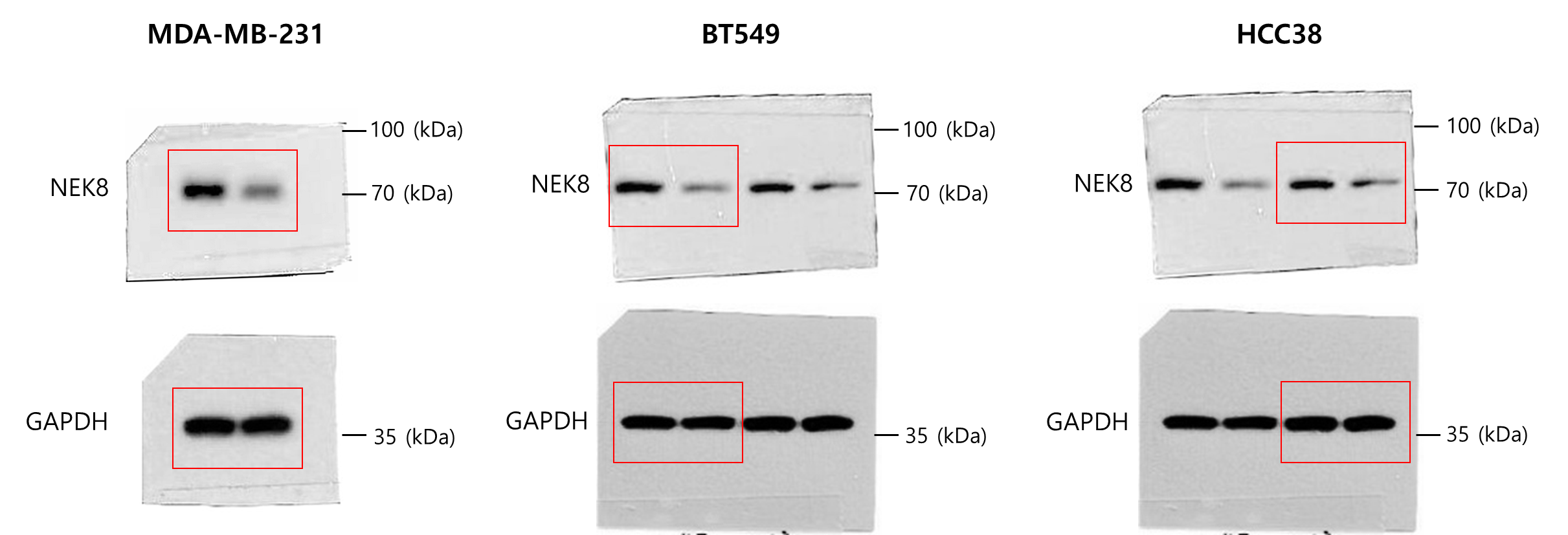
**

**Figure 2F**

**
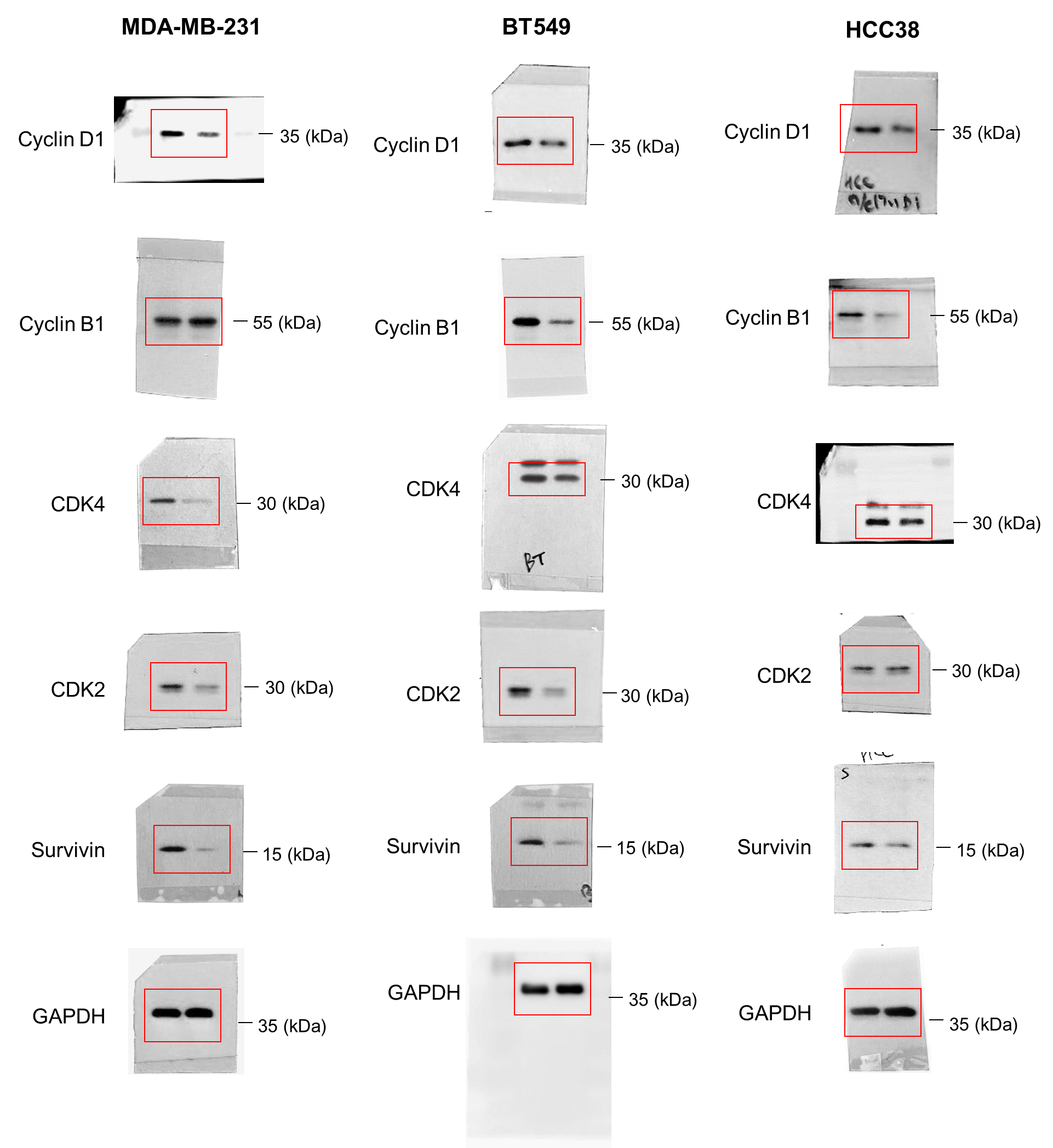
**

**Figure 3D**

**
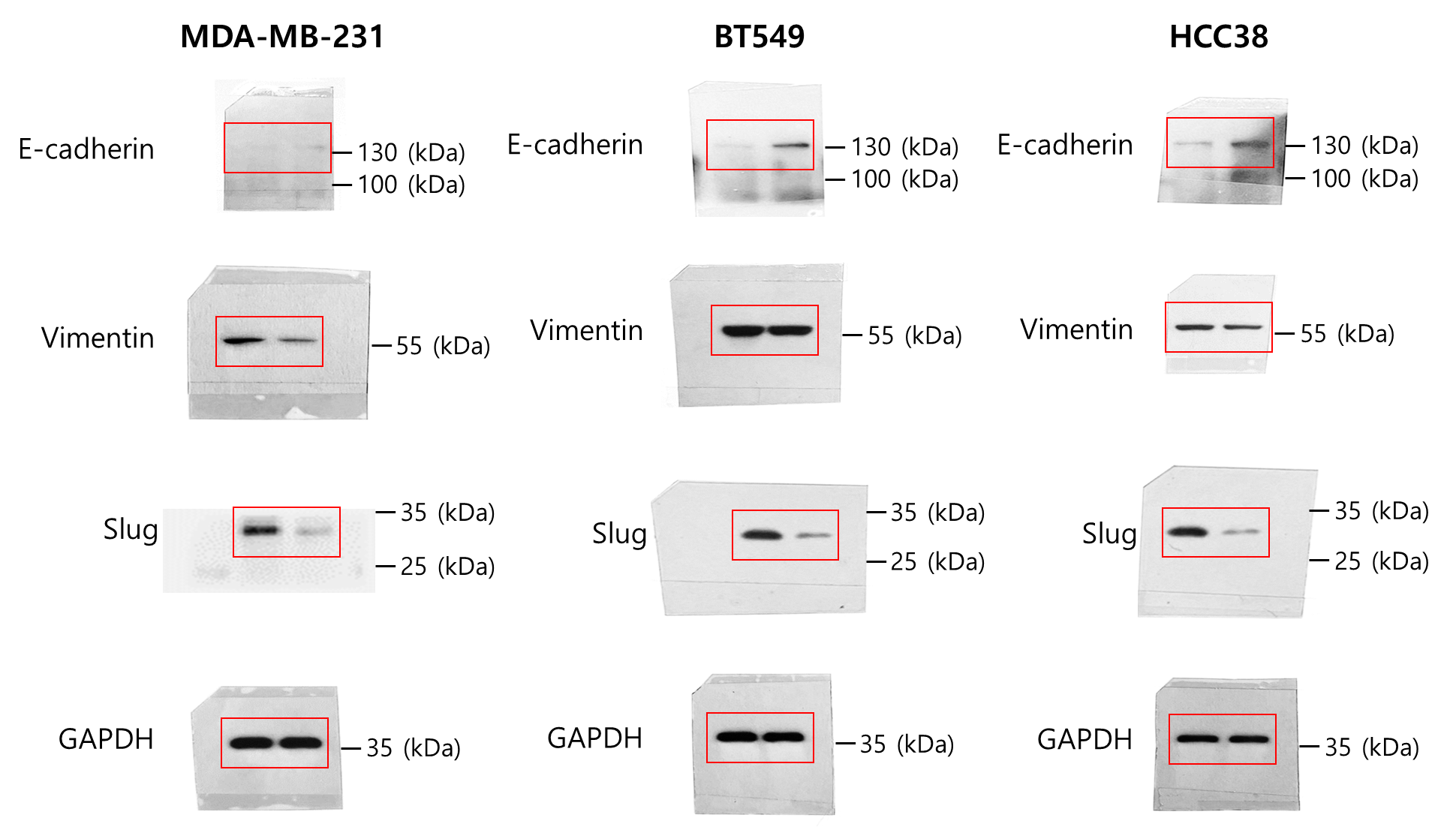
**

**Figure 4C**


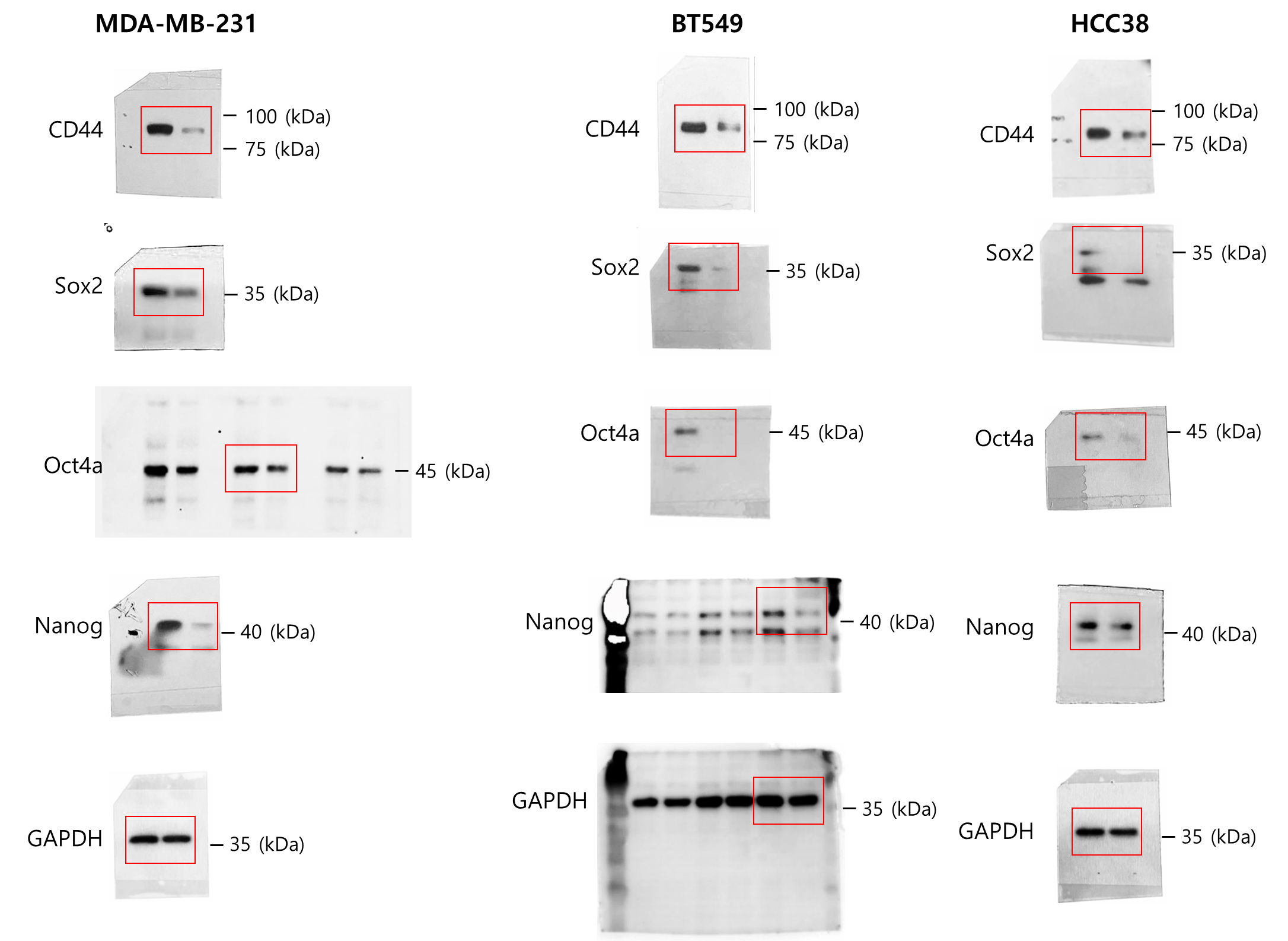


**Figure 5A**

**
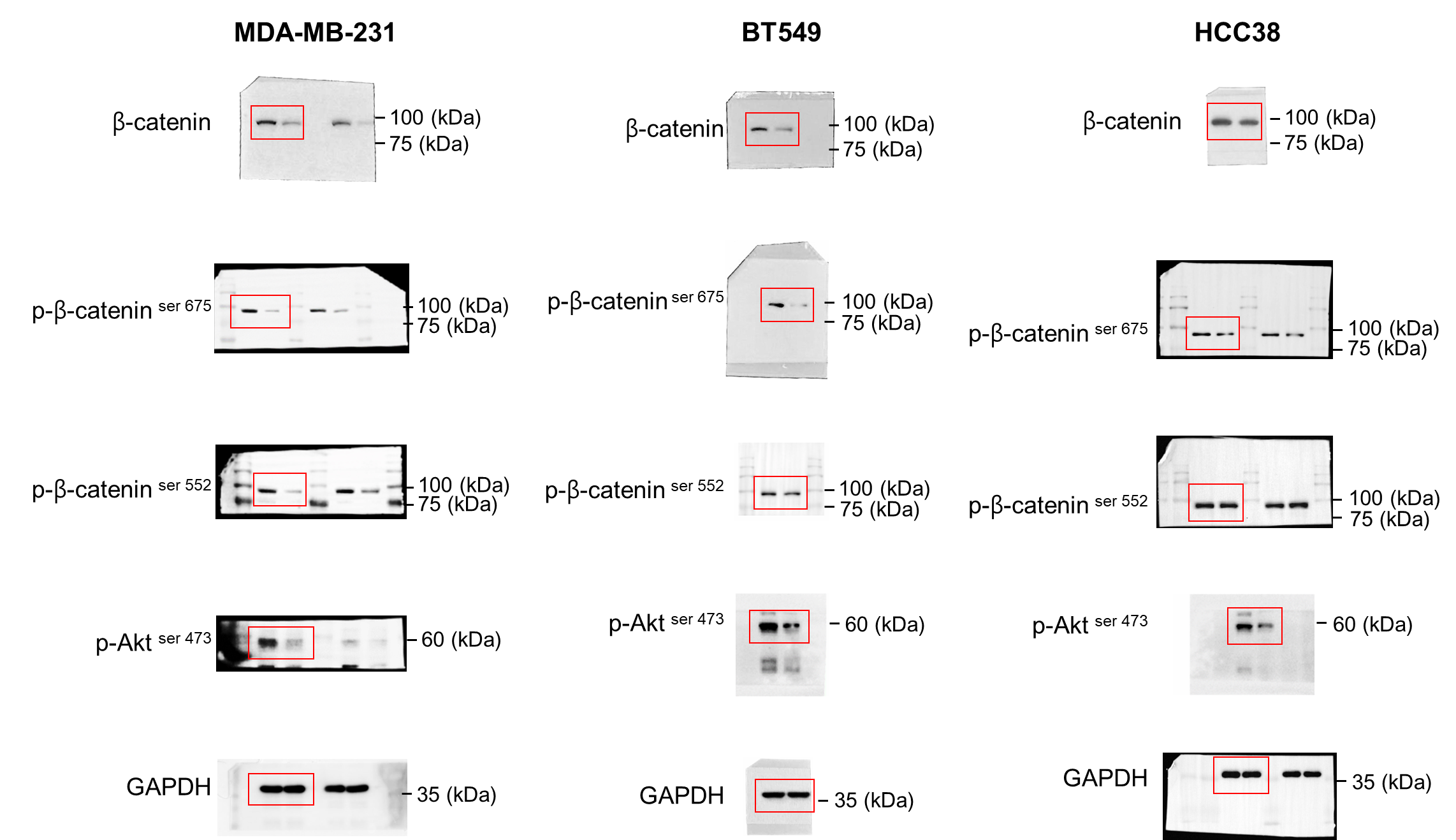
**

**
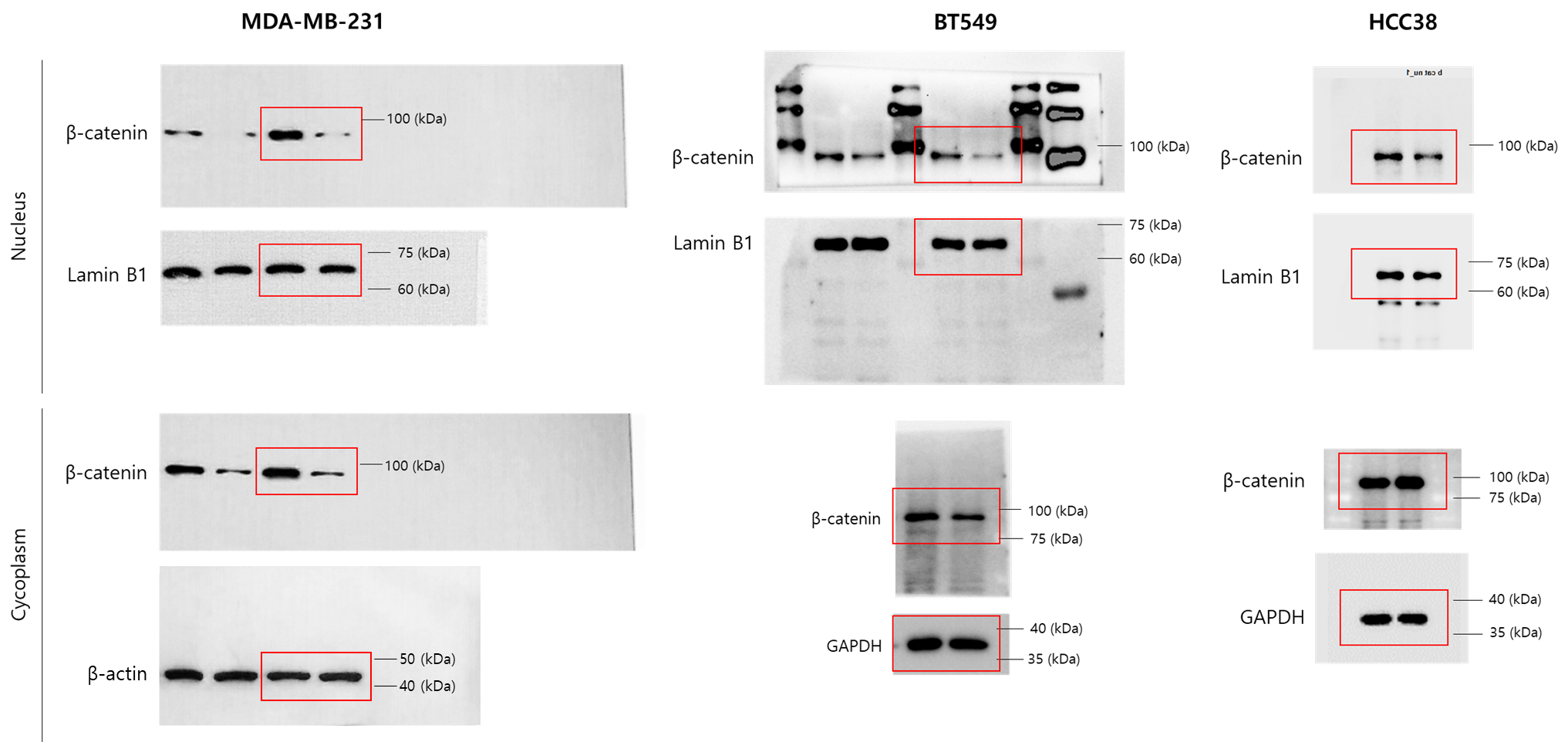
Figure 5B**

**Figure 5D**

**
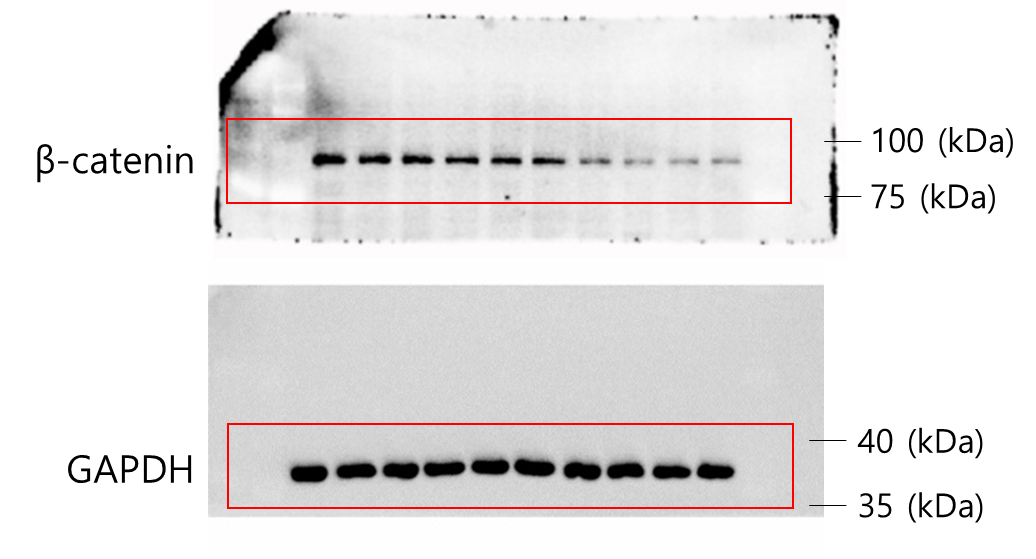
**

**Figure 5E**


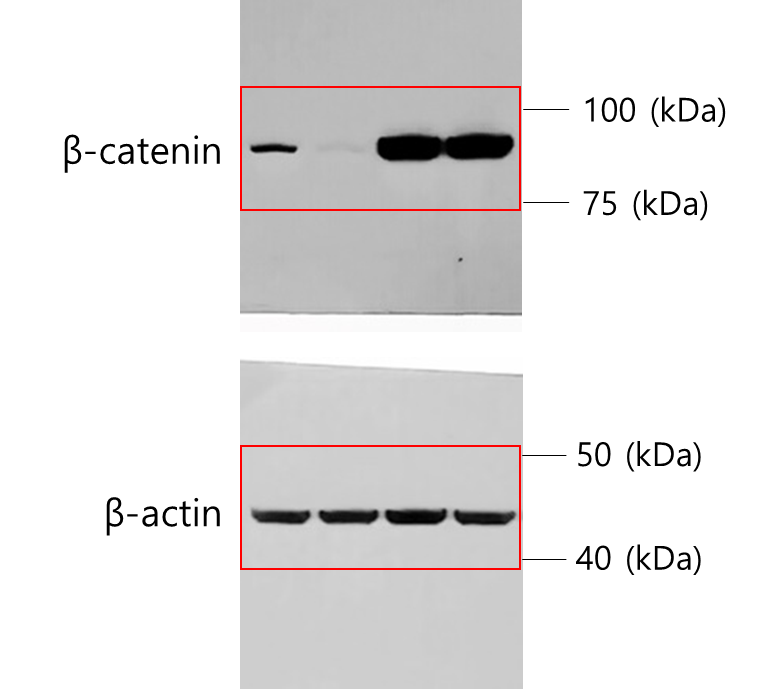


**Figure 5F**

**
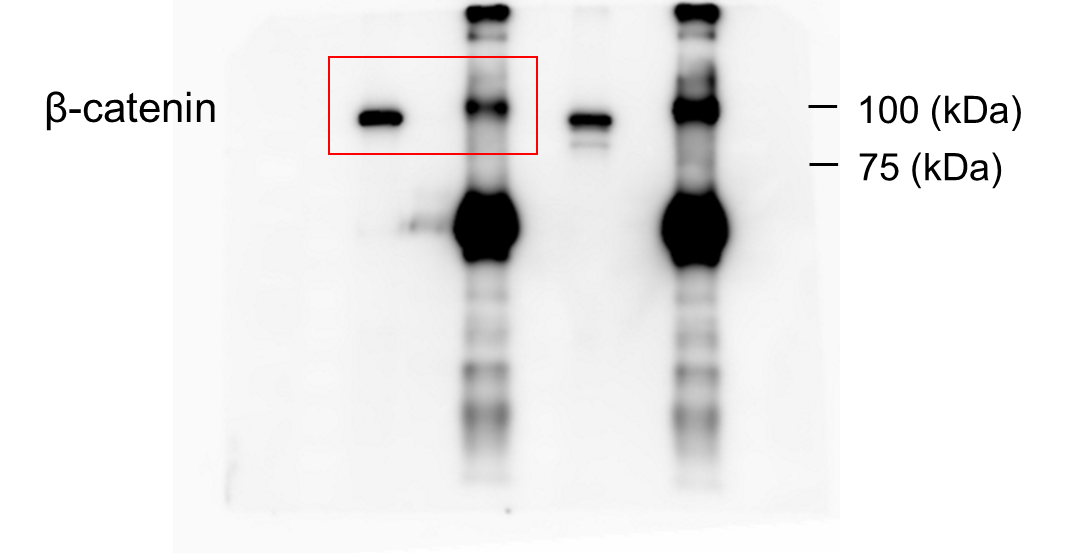
**

**Figure 6A**

**
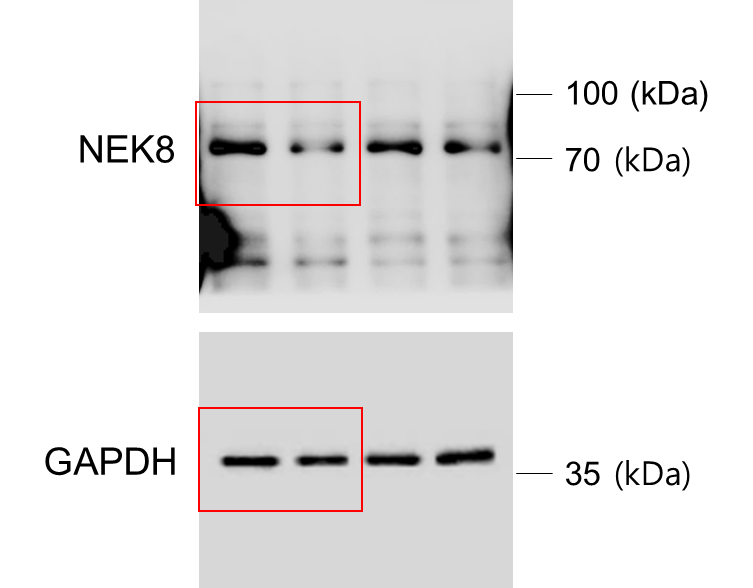
**

**Supplementary Figure S1**

**
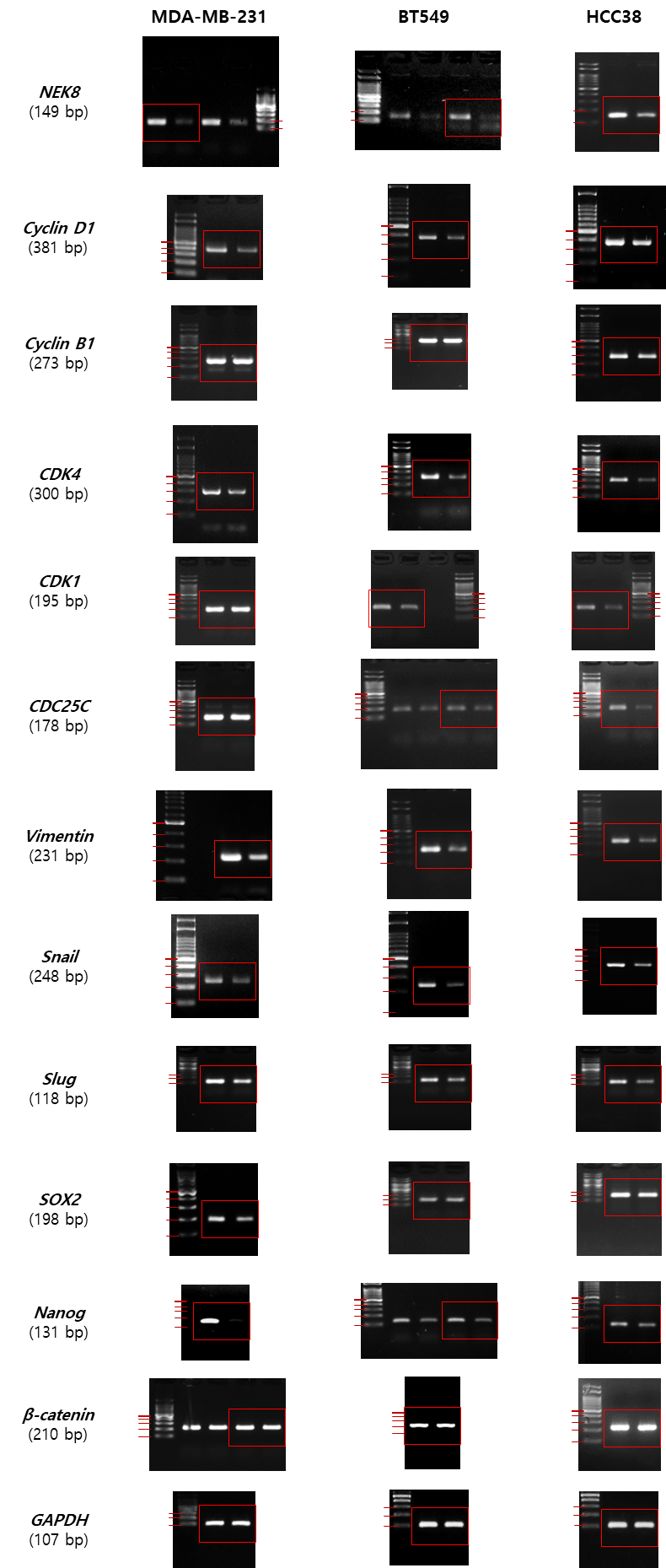
**
