# Supplementary material for: Never in mitosis gene A-related kinase-8 promotes proliferation, migration, invasion, and stemness of breast cancer cells via β-catenin signalling activation
Source: Sci Rep. 2023 Apr 26;13:6829. doi: 10.1038/s41598-023-32631-3 (PMC10133229; doi:10.1038/s41598-023-32631-3)
Supplement: Supplementary file 2 — Supplementary Tables. [file 41598_2023_32631_MOESM2_ESM.pdf]

Supplementary Table 1. siRNA sequences

1. L-004866-00, Human NEK8 siRNA – SMARTpool

1) ON-TARGETplus SMARTpool siRNA J-004866-05, NEK8

AGACAAAGCCCUUAUGAUC

2) ON-TARGETplus SMARTpool siRNA J-004866-06, NEK8

GUAAUUCCCUGCUGGAGGA

3) ON-TARGETplus SMARTpool siRNA J-004866-07, NEK8

GCGAAAGGCUGACCAGAAG

4) ON-TARGETplus SMARTpool siRNA J-004866-08, NEK8

GGGCAGAGAGCGAAGUGUA

2. D-001810-10, Non-targeting Pool

1) ON-TARGETplus Non-targeting Control siRNA D-001810-01

UGGUUUACAUGUCGACUAA

2) ON-TARGETplus Non-targeting Control siRNA D-001810-02

UGGUUUACAUGUUGUGUGA

3) ON-TARGETplus Non-targeting Control siRNA D-001810-03

UGGUUUACAUGUUUUCUGA

4) ON-TARGETplus Non-targeting Control siRNA D-001810-04

UGGUUUACAUGUUUUCCUA

Supplementary Table 2. shRNA sequences

sc-61176-V: Nek8 shRNA (h) Lentiviral Particles is a pool of 3 different shRNA plasmids:

all sequences are provided in 5′ → 3′ orientation.

1) sc-61176-VA:

Hairpin sequence:

GATCCGGCATCTCCAAGATCCTTATTCAAGAGATAAGGATCTTGGAGATGCCTTTTT

2) sc-61176-VB:

Hairpin sequence:

GATCCGTTGTATGTGGTATCGATTTTCAAGAGAAATCGATACCACATACAACTTTTT

3) sc-61176-VC:

Hairpin sequence:

GATCCCCTGAGCTTCACACTACTATTCAAGAGATAGTAGTGTGAAGCTCAGGTTTT
T

Supplementary Table 3. RT-PCR Primer sequences

Gene	Primer sequences (5' → 3')	Product length (bp)
NEK8	Forward: GCAAGCCCTACAACCAGAAG<br>Reverse: CCGGTCAGAGATAGGTGCAA	149
Cyclin D1	Forward: AAGGCGGAGGAGACCTGCGC<br>Reverse: ATCGTGCGGGGTCATTGCGG	381
CyclinB1	Forward: ACTGAGGCCAAGAACAGCTC<br>Reverse: GGGCTTGGAGAGGCAGTATC	273
CDK4	Forward: TGGCTTTACTGAGGCGACTG<br>Reverse: CCAGCTTGACTGTTCCACCA	300
CDK1	Forward: ACTAGAAAGTGAAGAGGAAGGG<br>Reverse: TCCATGTACTGACCAGGAGG	195
CDC25C	Forward: GTCTGCGTGAAGAGGACAG<br>Reverse: CTTCGACACCTCAGCAACTC	178
Vimentin	Forward: CTCTGGCACGTCTTGACCTT<br>Reverse: ACCATTCTTCTGCCTCCTGC	231
Snail	Forward: GAGGACAGTGGGAAAGGCTC<br>Reverse: TGGCTTCGGATGTGCATCTT	248
Slug	Forward: GGATCTCTGGTTGTGGTATG<br>Reverse: AAGCATTTCAACGCCTCCAA	118
SOX2	Forward: TTGTTCGATCCCAACTTTCC<br>Reverse: ACATGGATTCTCGGCAGACT	198
Nanog	Forward: GCAGATGCAAGAACTCTCCA<br>Reverse: GCGTCACACCATTGCTATTC	131
β-catenin	Forward: CCGACACCAAGAAGCAGAGA<br>Reverse: GCACGAACAAGCAACTGAAC	210
GAPDH	Forward: GAAGGGGTCATTGATGGCAA<br>Reverse: GGAAGGTGAAGGTCGGAGTC	107
