# Supplementary material for: Never in mitosis gene A-related kinase-8 promotes proliferation, migration, invasion, and stemness of breast cancer cells via β-catenin signalling activation
Source: Sci Rep. 2023 Apr 26;13:6829. doi: 10.1038/s41598-023-32631-3 (PMC10133229; doi:10.1038/s41598-023-32631-3)
Supplement: Supplementary file 3 — Supplementary Legends. [file 41598_2023_32631_MOESM3_ESM.doc]

**Supplementary Figure Legend**

**Supplementary Figure 1. Knockdown of NEK8 regulates proliferation, EMT and cancer stem cell-related genes.**

MDA-MB-231, BT549 and HCC38 cells were transfected with non-targeting (siCtrl) or NEK8 siRNA. After 48h, the mRNA levels of proliferation-related genes (*Cyclin D1, Cyclin B1, CDK4, CDK1, CDC25C*), EMT-related genes (*Vimentin, Snail, Slug*) and cancer stem cell-related genes (*SOX2, Nanog*) were measured through RT-PCR. *GAPDH* was used as a loading control. This figure is representative data from three independent experiments (n = 3). Error bars indicate SD; ^***^*P* < 0.001, ^**^*P* < 0.01, ^*^*P* < 0.05.
