# Supplementary material for: Never in mitosis gene A-related kinase-8 promotes proliferation, migration, invasion, and stemness of breast cancer cells via β-catenin signalling activation
Source: Sci Rep. 2023 Apr 26;13:6829. doi: 10.1038/s41598-023-32631-3 (PMC10133229; doi:10.1038/s41598-023-32631-3)
Supplement: Supplementary file 4 — Supplementary Figure S1. [file 41598_2023_32631_MOESM4_ESM.ppt]

## Slide 1
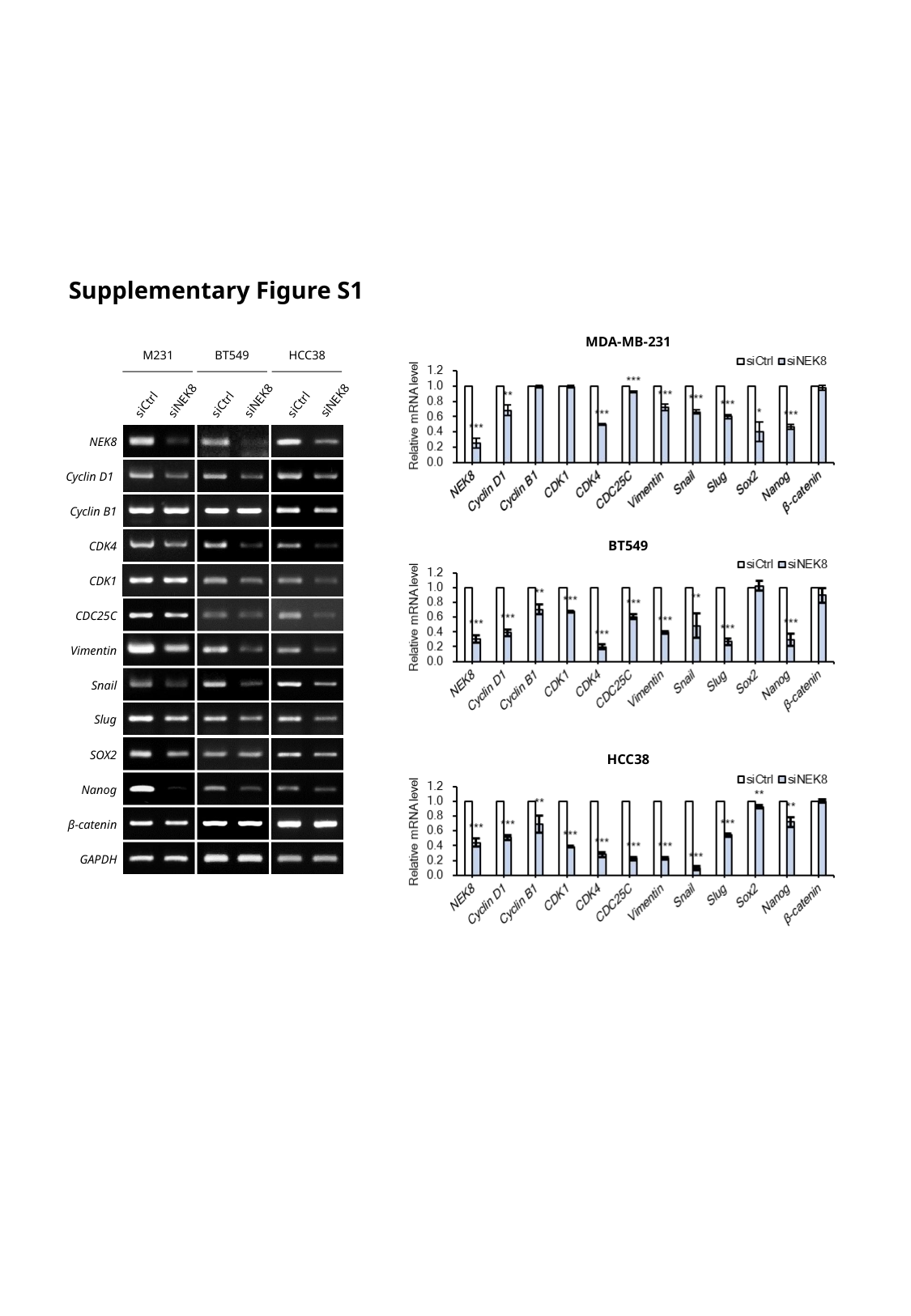

Supplementary Figure S1
MDA-MB-231
M231
BT549
HCC38
siNEK8
siNEK8
siNEK8
siCtrl
siCtrl
siCtrl
NEK8
Cyclin D1
Cyclin B1
BT549
CDK4
CDK1
CDC25C
Vimentin
Snail
Slug
SOX2
HCC38
Nanog
β-catenin
GAPDH
